# Analysis of Codon Usage Patterns in *Giardia duodenalis* Based on Transcriptome Data from *Giardia*DB

**DOI:** 10.3390/genes12081169

**Published:** 2021-07-29

**Authors:** Xin Li, Xiaocen Wang, Pengtao Gong, Nan Zhang, Xichen Zhang, Jianhua Li

**Affiliations:** Key Laboratory of Zoonosis Research, Ministry of Education, College of Veterinary Medicine, Jilin University, Changchun 130062, China; lixin2018@jlu.edu.cn (X.L.); wangxiaocen20@jlu.edu.cn (X.W.); gongpt@jlu.edu.cn (P.G.); n_zhang@jlu.edu.cn (N.Z.); xczhang@jlu.edu.cn (X.Z.)

**Keywords:** *Giardia duodenalis*, codon usage bias, transcriptome, optimal codon, evolution

## Abstract

*Giardia duodenalis*, a flagellated parasitic protozoan, the most common cause of parasite-induced diarrheal diseases worldwide. Codon usage bias (CUB) is an important evolutionary character in most species. However, *G. duodenalis* CUB remains unclear. Thus, this study analyzes codon usage patterns to assess the restriction factors and obtain useful information in shaping *G. duodenalis* CUB. The neutrality analysis result indicates that *G. duodenalis* has a wide GC3 distribution, which significantly correlates with GC12. ENC-plot result—suggesting that most genes were close to the expected curve with only a few strayed away points. This indicates that mutational pressure and natural selection played an important role in the development of CUB. The Parity Rule 2 plot (PR2) result demonstrates that the usage of GC and AT was out of proportion. Interestingly, we identified 26 optimal codons in the *G. duodenalis* genome, ending with G or C. In addition, GC content, gene expression, and protein size also influence *G. duodenalis* CUB formation. This study systematically analyzes *G. duodenalis* codon usage pattern and clarifies the mechanisms of *G. duodenalis* CUB. These results will be very useful to identify new genes, molecular genetic manipulation, and study of *G. duodenalis* evolution.

## 1. Introduction

Codon usage bias is a widespread feature among both prokaryotes and eukaryotes, which describes synonymous codons that are not used at the same frequency in the process of gene translation [1,2,3]. CUB is widely distributed and affected by tRNA abundance [4], nucleotide composition [5], translational processes [6], gene function [7,8], protein structure [9], hydrophobicity [10], environment temperature [11], etc. Among the known factors affecting CUB in different organisms, constraints on composition and translation selection are believed to be the primary causes for the differences in CUB among genes in different species [12]. CUB can be used to optimize the expression of foreign genes in given host cells. In addition, CUB could also provide clues about the evolution and environmental adaptation of various species [13].

Analysis of synonymous codon usage bias has various important applications, such as degenerate primer design [14], heterologous gene expression [15], species origin determination [16], the prediction of the expression level of genes [17,18], and the prediction of gene functions [19]. CUB has been studied in various species, such as *Taenia saginata* [20], *Taenia multiceps* [21], *Plasmodium falciparum* [22], *Entamoeba histolytica* [23], *Microsporidia* [24], *Caenorhabditis*, *Drosophila*, *Arabidopsis* [25], *Streptomyces* [26], *Borrelia burgdorferi* [27], and *Saccharomyces cerevisiae* [28,29]. *Giardiasis* is a worldwide epidemic water source diarrhea disease, which can infect various mammals, including human beings [30,31,32]. Previous studies of *G. duodenalis* codon usage only analyzed with small number of genes [33,34].

This study investigates the CUB of *G. duodenalis* from transcriptome data, which provides useful information for elucidation of the mechanism of synonymous CUB, design of gene vaccine, and better control strategy of *G. duodenalis*.

## 2. Methods

### 2.1. Transcriptome Data

Nine thousand, seven hundred and forty-seven coding sequences (CDS) of *G. duodenalis* (*Giardia* Assemblage A isolate WB) obtained from GiardiaDB were investigated based on the transcriptome profiling of Oscar Franzén et al. [35]. To minimize outliers caused by small size, CDS with sizes below 300 bp were eliminated. Thus, a total of 5968 CDS were selected for the analysis.

### 2.2. Indices of Codon Usage

A codon usage table of *G. duodenalis* was obtained from Codon Usage Database via Optimizer software [36]. CDS sequences codon usage of *G. duodenalis* genes was analyzed by Codon W 1.4.4 software and EMBOSS online tools (https://www.bioinformatics.nl/emboss-explorer/, accessed on 26 July 2021). 17 CDS sequences codon usage of *G. duodenalis* highly expressed variant-specific surface proteins (VSPs) [37] were analyzed by EMBOSS online tools (https://www.bioinformatics.nl/emboss-explorer/, accessed on 26 July 2021). The analysis parameters included an effective number of codons (ENC), the codon adaptation index (CAI), relative synonymous codon usage (RSCU), GC content (guanine and cytosine content), A3s, T3s, G3s, C3s, and GC3s. Nucleotide composition and its frequency on the third synonymous codon can be used to reflect the codon usage preference of CDS sequences. The RSCU value is the observed codon frequency divided by the frequency expected under the same assumption for amino acid synonymous codons, and it is an index for studying differences in synonymous codon usage among genes. The RSCU values were measured according to the previous study [12]. ENC values reflect the number of codon types used in a gene, and its value generally ranged from 20 (when only one codon was used) to 61 (when all codons are used equally), and the ENC values were measured as previously described [38]. CAI refers to the consistency between the usage frequency of synonymous codons and optimal codons in the coding region, with a value of 0–1. CAI is used to estimate the degree of CUB, which is preferred in highly expressed genes. Higher CAI values mean that CUB may be stronger, and the potential expression level may be higher, and the CAI values were calculated as previously described [39].

### 2.3. Neutrality Plot

The effect of selection on CUB was usually measured by neutrality plot analysis. In this method, the average GC content of GC12 and GC3s was calculated. The scatter plot was drawn with GC3s as an independent variable and GC12 as a dependent variable. The points representing genes are distributed on or near the diagonal, indicating that the codon usage pattern is greatly affected by mutation; on the contrary, the smaller the slope of the scatter formation curve is, or even parallels to the horizontal axis, indicating that codon usage pattern is greatly influenced by environmental selection [40].

### 2.4. ENC Plot

The ENC plot of ENC values plotted against GC3s values is used to analyze the influence of base composition on the codon usage in a genome [41]. A standard curve is used to show the functional relationship between ENC and GC3s values under mutation pressure rather than selection pressure. In the ENC plot correlation analysis, GC3s were used as the independent variable and ENC as the dependent variable to construct the scatter diagram and to analyze the correlation between ENC and GC3s. In addition, according to the CUB, the standard curve was constructed under the condition of mutation pressure, but not selection pressure. If the predicted ENC value is on or near the standard curve, it represents that the CUB is mainly influenced by mutation rather than selection pressure; if the predicted ENC value is far below the standard curve, it represents that the codon composition is mainly affected by selection pressure [41].

### 2.5. PR2 Bias Plot Analysis

Parity rule 2 analysis, also known as parity rule analysis, is a method to study the base composition of codons. If the gene is not under the pressure of mutation or environmental selection, the internal composition of the base is A = T, C = G. However, due to the influence of gene mutation and environmental selection pressure, the usage of G and C in the genome coding sequence is often uneven, especially the third codon deviates from the rule of intrachain equivalence. In this method, amino acids encoded by four synonymous codons were analyzed, and the calculated results of G3/ (G3 + C3) and A3/ (A3 + T3) were plotted. The coordinates (0.5, 0.5) represent the PR2 principle (A = T, C = G). The distance and position of the scattered points from the center indicate the degree and direction of the gene deviation from the rules [42].

### 2.6. Determination of Optimal Codons

Based on the CAI values, 5% of the total genes with extremely high and low CAI values were regarded as high and low datasets, respectively. Codon usage was compared using a Chi-squared contingency test of the two groups, and codons whose frequency of usage was significantly higher (*p* < 0.01) in highly-expressed genes than those with low levels of expression were defined as optimal codons [43].

### 2.7. Correspondence Analysis (COA)

The connection between variables and samples was widely analyzed by Multivariate statistical analysis. COA has been widely used to study codon usage variation. CondonW was used to analyze RSCU values by COA analysis, COA was performed on RSCU values using to compared the intra-genomic variation of 59 informative codons partitioned along 59 orthogonal axes (excluding Met, Trp, and stop codons) [44].

### 2.8. Statistical Analysis

The indices of codon usage were analyzed by CondonW1.4.4 software. Microsoft Excel and SPSS 19.0 were used to analyze the correlation based on Spearman’s rank correlation.

## 3. Results

### 3.1. Nucleotide Contents of G. duodenalis Genes

**Figure 1:**
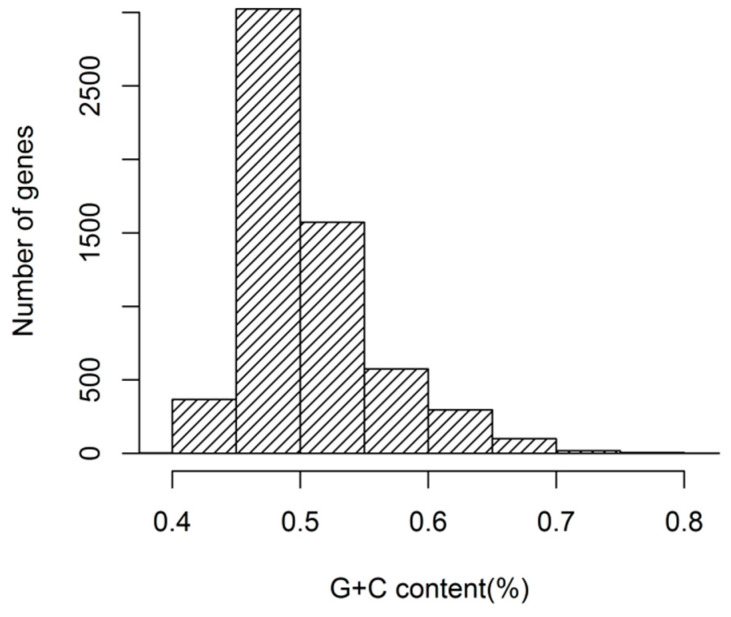
Distribution of GC contents in *G. duodenalis* genes.

The nucleotide contents of *G. duodenalis* CDSs (expressed as % GC) were shown in Figure 1. The results suggested that the GC content of 5968 *G. duodenalis* genes exhibited a distinctly unimodal distribution. The GC contents of *G. duodenalis* genes varied from 39.1% to 81.1% (SD = 0.0523), and the GC contents of the 5968 CDSs were mainly ranged from 45% to 55%. To understand the distribution of nucleotides, we studied the content of GC and GC3s. GC1, GC2 and GC3 were 53.38%, 41.79% and 51.67%, respectively. This result suggested that the GC2 was different from GC1, and GC3—GC2 was the lowest among the three codon positions. The mean value of GC contents of all codons was 48.95%.

**Figure 2:**
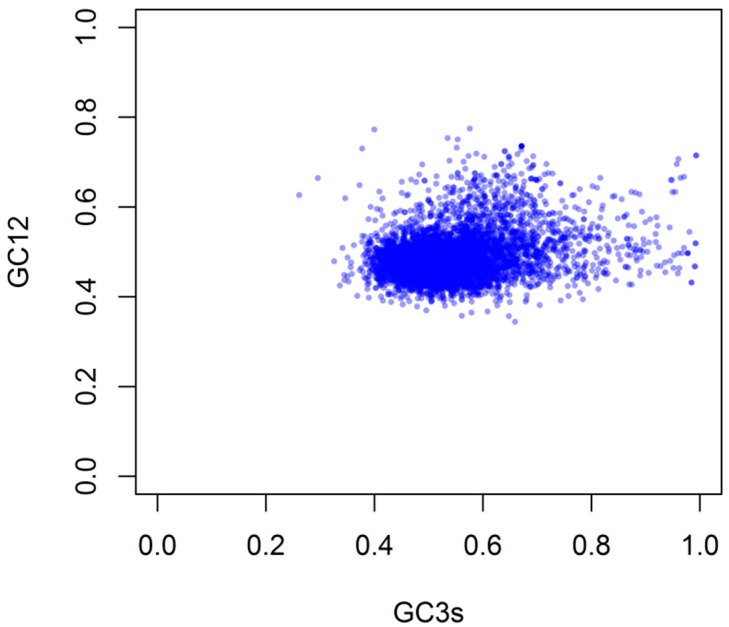
Neutrality plots analysis of GC12 and GC3 of *G. duodenalis* transcriptome.

To study the relationship between the three codon positions, the neutrality graph (GC12 versus GC3) of the *G. duodenalis* gene was constructed. Our result demonstrated that the GC3s of *G. duodenalis* genes was widely distributed (26.17% to 99.32%), and there was a significant correlation between GC12 and GC3 (r = 0.283, *p* < 0.0001) (Figure 2), indicating that mutation might affect the codon preference in *G. duodenalis* genome.

### 3.2. Codon Usage in G. duodenalis

The synonymous codon usage patterns of *G. duodenalis* were shown in Table 1. The G + C content of the *G. duodenalis* genome was 49.1%, which indicated that the *G. duodenalis* genome was a little AT-rich. The total codon usage was biased towards G-and C-terminal codons (31 codons were common codons, and 16/31 frequently used codons ended with G or C). These results indicated that gene composition restriction played a significant role in the formation of codon usage variation in the *G. duodenalis* genome. In addition, we analyzed 17 CDS sequences codon usage of *G. duodenalis* highly expressed VSPs genes based on the actual protein levels [37], and 12/15 most frequently used codons were biased towards G-and C-terminal codons (Table 2). At last, a codon usage table of *G. duodenalis* was obtained from Codon Usage Database via Optimizer software, and 9/10 most frequently used codons were biased towards G-and C-terminal codons (Table 3). These results further confirm the accuracy of our predicted codon usage bias.

### 3.3. Relation between ENC and GC3

**Figure 3:**
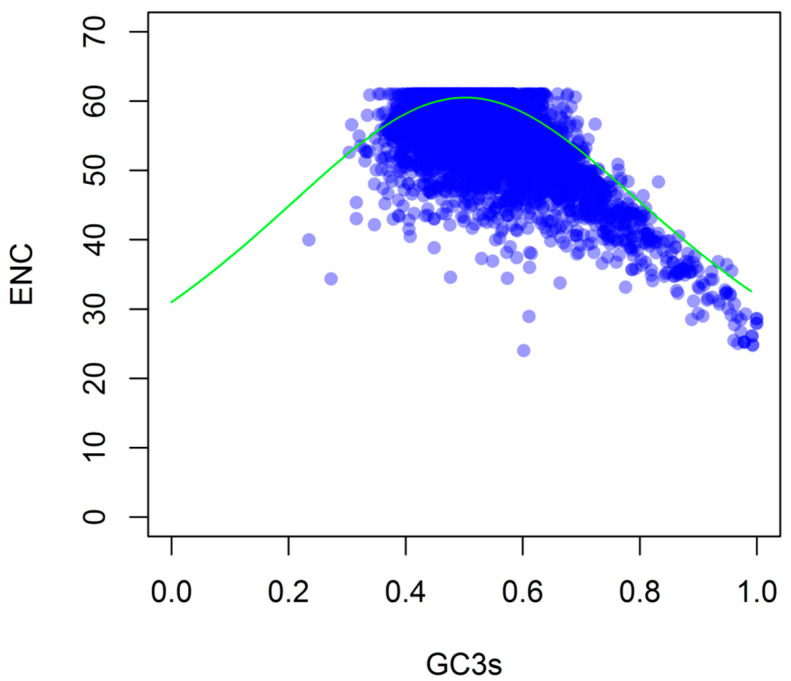
Distribution of ENC and GC3s of *G. duodenalis* genes. ENC is plotted against GC3s. The solid line (green) shows the expected ENC value if the CUB is caused by GC3s only.

**Figure 4:**
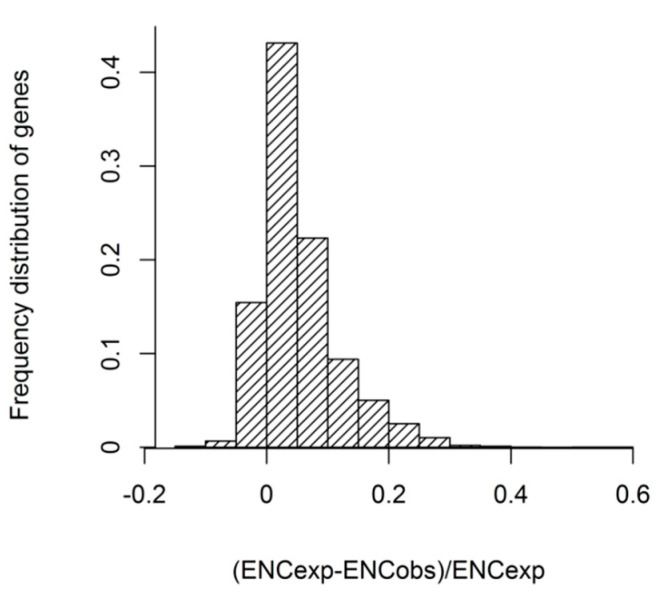
Frequency distribution of the ENC ratio.

ENC values were analyzed according to the fraction of GC3s (Figure 3) to clarify the relationship between nucleotide composition and *G. duodenalis* CUB. The ENC values ranged from 24 to 61, suggesting significant differences in codon bias among these genes. As shown in Figure 3, most of the points were clustered near the expected ENC curve, which indicated that the ENC of most genes was close to the expected ENC value based on their GC3. In addition, the ENC of some points was lower than the expected curve, indicating that the codon usage was also affected by other factors beyond mutation pressure.

(ENCexp-ENCobs)/ENCexp was calculated to estimate the observed and expected ENC values more accurately. We found that (ENCexp-ENCobs)/ENCexp was mainly located in 0–0.05, and the values of most genes were located in −0.05–0.15 (Figure 4). The results suggested that the ENCs of most genes were slightly different from the expected ENCs values in GC3s. The observed ENCs values of most genes were near expected ENCs in GC3s, although the ENCs values of some genes were much lower.

### 3.4. Correspondence Analysis

**Figure 5:**
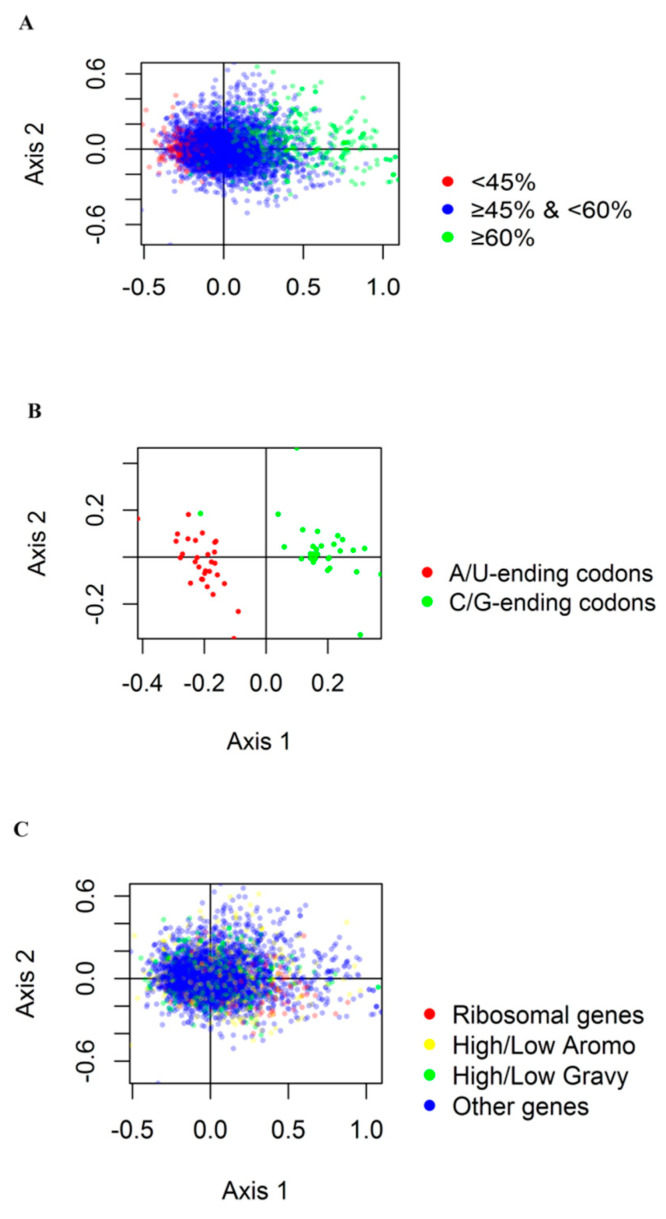
Correspondence analysis of RSCU for *G. duodenalis* genes. (**A**). The first two axes show the distribution of *G. duodenalis* genes. Green dots represent G + C content ≥ 60%; Blue dots represent G + C content ≥ 45%, but less than 60%; Red dots represent G + C content ≤ 45%. (**B**). Panel A shows the distribution of codons on the same two axes. Red dots indicate A and T ending codons, and green dots indicate G and C ending codons. (**C**). Red dots represent ribosomal genes, yellow dots represent genes with an Aromo value ≥0.15, green dots represent genes with a Gravy value higher than 5, and blue dots represent other genes.

The differences in synonymous codon usage among *G. duodenalis* genes were further studied by RSCU correspondence analysis. We determined four major contributors as axis 1–4. Axis 1 and axis 2 accounted for 14.81% and 5.30% of the total variance, respectively, while axis 3 and axis 4 accounted for 4.05% and 3.46% of the total variance, respectively, suggesting that axis 1 and axis 2 were the main contributors to *G. duodenalis* CUB. Axis 1 and axis 2 showed each gene in Figure 5A. To clarify the effect of gene GC contents on CUB, the gene GC contents were color coded. The genes with GC content more than or equal to 60% were shown in green, while those with GC content less than 45% were shown in red. GC content ranged from 45% to 60% were shown in blue. The results showed that the high and low GC contents of *G. duodenalis* genes could be separated by the primary axis. In addition, as shown in Figure 5B, we found that the terminal codons of different bases could be separated along two axes. It seemed that the separation of the first axis codon was mainly due to the frequency difference of G/C and A/T terminal codons. Further calculation showed a significant correlation between the GC content of each gene and its position on the first axis (r = 0.1154, *p* < 0.0001). The location of axis 1 gene was positively correlated with GC3s (r = 0.1160, *p* < 0.0001) and negatively correlated with ENC (r = −0.0626, *p* < 0.0001). These results indicated that the genes with higher GC and GC3 contents and lower ENC content on the left side of the first axis showed stronger codon bias, which demonstrated that the main factor affecting the CUB of *G. duodenalis* was the nucleotide composition.

To investigate different gene codon usages, we selected hydrophobic genes, aromatic genes, ribosomal genes, and other genes from 5968 genes. Figure 5C showed the distribution of these four categories of genes. Multivariate analysis of variance indicated that there were statistically significant differences in codon usage among these different genes (*p* < 0.01).

### 3.5. PR2-Bias Plot Analysis

**Figure 6:**
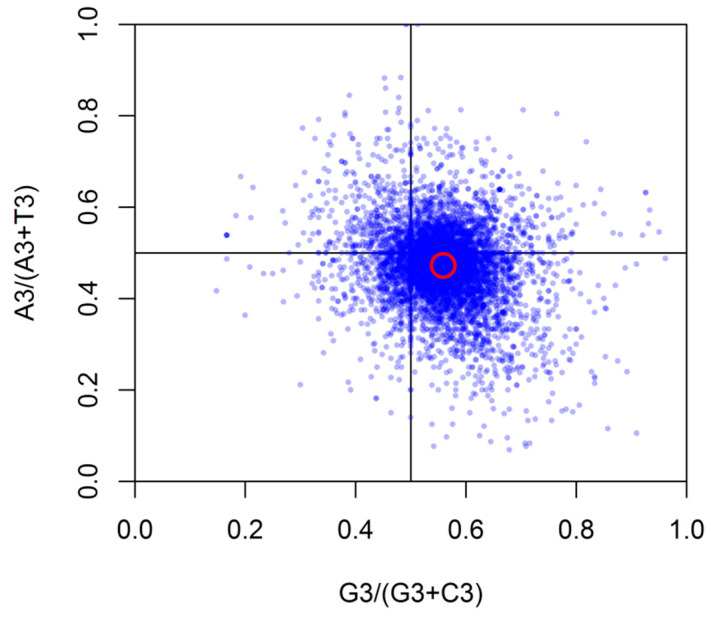
PR2-bias plot [A3/(A3 + T3) against G3/(G3 + C3)]. Red circle represents the average position for each plot.

To study whether high bias genes restrict the selection of biased codons, the relationship between A/G purines and C/T pyrimidines in amino acid was analyzed by PR2 bias plot. Figure 6 showed that genes in the upper left quadrant had low expression and the genes in the lower right had high expression. We demonstrated that *G. duodenalis* preferred to use G and T rather than use C and A (Figure 6), which suggested that mutation bias, selection, and other factors were involved in *G. duodenalis* codon usage bias.

### 3.6. Role of Gene Expression Level and Encoded Protein Size Synonymous CUB

**Figure 7:**
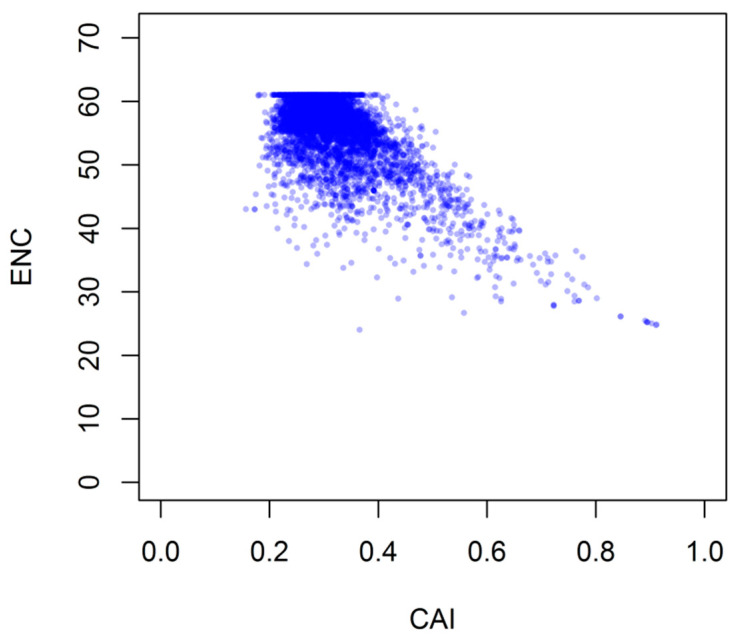
Relationship between ENC and gene expression level for.

To investigate the relationship between CUB and gene expression level, the correlation coefficient between CAI and ENC was calculated and analyzed. The CAI values were used to evaluate *G. duodenalis* genes expression level, which ranged from 0.157 to 0.912 (mean = 0.318, SD = 0.07942). As shown in Figure 7, our results indicated that genes expression level were significantly negatively correlated with genes position along axis 1 (r = 0.6811, −0.1086, *p* < 0.0001), while CAI value showed positive relationship significantly with GC3s and GC content (r = 0.9022, 0.6486, respectively, *p* < 0.0001). These results demonstrated that highly expressed genes had a strong CUB and preferred to choose G or C codons in synonymous positions.

**Figure 8:**
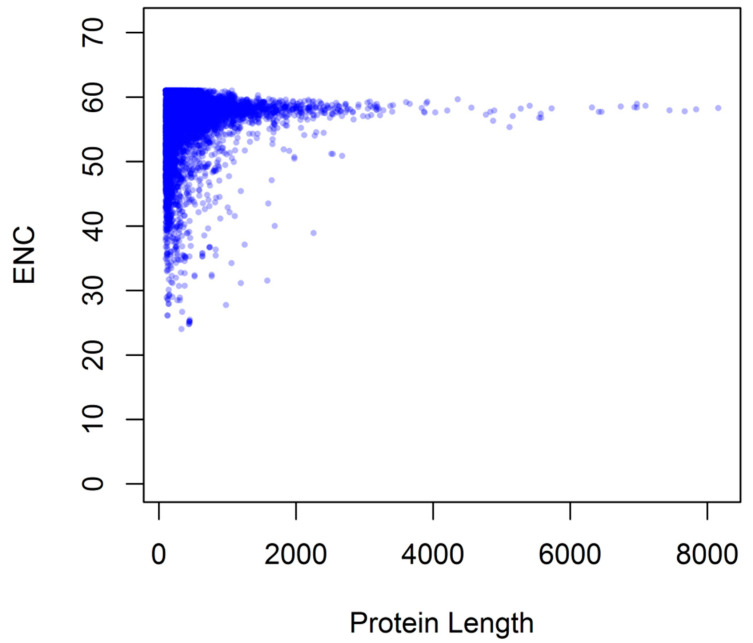
Relationship between ENC and encoded protein length for *G. duodenalis*.

As shown in Figure 8, correlation analysis between protein size and axis 1 values indicated that the 3 correlation coefficients (r = −0.0817, 0.1822, −0.1254, respectively, *p* < 0.01) all significantly correlated, suggesting that genes with higher expression levels had a smaller size.

### 3.7. Optimal Codons

Translational optimal codons of *G. duodenalis* were represented by the average value of RSCU in high/low expressed genes (Table 4). Chi-square test showed that 26 codons were optimal codons, and the frequency of these codons was significantly higher in highly expressed genes (*p* < 0.01), which all end in G or C, indicating that *G. duodenalis* preferred to use G or C ending synonymous codons.

## 4. Discussion

CUB widely exists among both prokaryotes and eukaryotes. This is an interesting and complex phenomenon in the process of biological evolution. Previous studies have proposed hypotheses trying to explain the origin of CUB, among which the selection-mutation-drift balance model and the neutral theory are the most influential ones [12,45], which considers that CUB is determined by the balance between mutation pressure, genetic drift, and weak selection [12,46], while neutral theory believes that mutations of degenerate coding cites should be neutral selection, which leads to random synonymous codon usage selection [45]. However, studies have also suggested that many others factors could affect CUB, such as GC-content [47,48], gene size [25], gene expression level [25,49,50], and gene recombination rate [47,49,51,52]. Furthermore, RNA and protein structure [41,53,54,55], intron length [56], evolutionary age of the genes [57], population size [58], the aromaticity and the hydrophobicity of the coding proteins [10,59] have all been found to be influencing factors.

A previous analysis of *G. duodenalis* codon usage was restricted, which only considered eight genes, and yet it seems that the codon usage of *G. duodenalis* has been quite heterogeneous [33]. Another analysis of *G. duodenalis* codon usage investigated 65 genes, and 21 codons were the optimal codons, which were all end in C or G and almost exclusively used in the highly expressed genes [34], which was similar to our conclusion. However, the CUB of *G. duodenalis* has not been fully studied yet. In the present study, we performed a more comprehensive analysis based on the whole transcriptome data and found that multiple factors influence shaping *G. duodenalis* CUB, such as mutation pressure, selection, gene expression, and compositional constraints, and protein size.

Generally speaking, nucleotide composition is one of the most important effect factors in forming codon usage, while GC content reflects the overall trend of codon mutation [60]. Some GC-rich organisms, such as Triticum Aestivum, Oryzasativa, Bacteria, Archea, and Fungi [61,62], have been proved that they tend to use G or C ending codons. In contrast, some AT-rich organisms, such as *Onchocerca volvulus*, *Mycoplasma capricolum*, and *P. falciparum* [63,64,65], have been shown that they tend to A or T ending codons. In this study, we demonstrated that the average GC content among the 5968 *G. duodenalis* genes was 49.1%. Although the genome of *G. duodenalis* seems to be slightly AT-rich, the usage of all codons is biased towards G or C-terminal codons (Table 2), which is similar to those in *T. saginata* [20] and *T. multiceps* [21].

Previous studies have demonstrated that over-expressed genes are expressed more frequently, and produced more protein than other genes [22]. Moreover, preferred codons were more frequently used in highly expressed genes than other genes [66]. ENC represents the species independent synonymous bias in genes [38,67]. The genes expression level of protein coding genes can be classified according to ENC values. Highly expressed genes show less ENC value, while the lowly expressed genes show more ENC value. In the present study, the ENC values of *G. duodenalis* genes ranged from 24–61, suggesting that the codon bias among these genes was significantly different.

CAI is a method of recognizing differential gene expression by codon selection. Highly expressed genes exhibit a high tendency to use certain codons and tend to use them frequently [68,69]. In the present study, the low CAI of *G. duodenalis* indicated that highly expressed genes faced greater translation selection pressure in shaping CUB, which is similar to the codon usage in *T. saginata* [20] and *P. falciparum* [70].

It has been confirmed that various organisms, such as *T. multiceps* [21], *T. saginata* [20], *S. cerevisiae* [29], *Silenelatifolia* [71], *Caenorhabditis elegans* [25], and *Arabidopsis thaliana* [25], showed significant negative correlations between gene size and CUB. Interestingly, our research suggested that *G. duodenalis* also showed a negative correlation between gene size and CUB, suggesting that selection restriction made *G. duodenalis* tend to produce smaller proteins with similar functions to larger proteins, thus reducing the energy consumption of producing specific functional proteins [72].

Identifying the optimal codons could provide an effective means for rational codon usage rearrangement and evolutionary molecular genetics research [73,74,75]. The optimal codons tend to reflect the GC and AT content of the genomes [62,76]. In this study, 26 codons were identified as optimal codons, ending with G or C. This phenomenon is similar to previous study of *G. duodenalis* [34] and other eukaryotic genomes, such as *T. multiceps* [21] and *T. saginata* [20]. Identifying optimal codons in the *G. duodenalis* genome might provide valuable information for genetic engineering and evolutionary study.

Our study revealed the pattern of CUB in the *G. duodenalis* genome and its influencing factors. Our results indicated that the CUB of *G. duodenalis* seemed to be a complex equilibrium under different pressures: Natural selection, mutation, GC content, gene expression level, and protein size. Interestingly, all 26 optimal codons ended with G or C, which would be useful for cloning and expression of foreign genes in *G. duodenalis*. Together, our study elucidated the codon usage pattern of *G. duodenalis* and provided useful information for genetic engineering and evolutionary studies in this primitive eukaryote.

## 5. Conclusions

This study systematically analyzes *G. duodenalis* codon usage pattern and clarifies the mechanisms of *G. duodenalis* CUB, which will be very useful to identify new genes, molecular genetic manipulation, and study of *G. duodenalis* evolution.

## Figures and Tables

**Table 1:** Codon usage in *G. duodenalis*.

AA	Codon	N	RSCU	AA	Codon	N	RSCU
Phe	**UUU**	59,550	1.07	Ser	**UCU**	73,318	1.49
	UUC	52,173	0.93		UCC	48,316	0.98
Leu	UUA	31,152	0.55		UCA	44,668	0.91
	UUG	46,111	0.82		UCG	32,496	0.66
	**CUU**	82,286	1.47	Pro	**CCU**	41,755	1.11
	**CUC**	68,932	1.23		CCC	36,359	0.97
	CUA	44,031	0.78		**CCA**	43,775	1.17
	**CUG**	64,305	1.15		CCG	28,158	0.75
Ile	**AUU**	64,266	1.06	Thr	**ACU**	51,120	1.01
	**AUC**	62,851	1.04		ACC	48,607	0.96
	AUA	54,849	0.90		**ACA**	64,162	1.27
Met	AUG	71,824	1.00		ACG	38,778	0.77
Val	**GUU**	53,711	1.12	Ala	**GCU**	66,007	1.04
	**GUC**	53,666	1.12		**GCC**	64,537	1.02
	GUA	33,623	0.70		**GCA**	82,061	1.29
	**GUG**	50,286	1.05		GCG	41,021	0.65
Tyr	**UAU**	53,440	1.01	Cys	UGU	32,743	0.82
	UAC	52,143	0.99		**UGC**	46,757	1.18
His	CAU	35,269	0.90	Arg	CGU	26,186	0.90
	**CAC**	43,263	1.10		**CGC**	35,590	1.22
Gln	CAA	50,096	0.79		CGA	22,041	0.76
	**CAG**	76,057	1.21		CGG	24,910	0.85
Asn	AAU	61,610	0.97	Ser	AGU	38,408	0.78
	**AAC**	66,024	1.03		**AGC**	57,389	1.17
Lys	AAA	50,623	0.63	Arg	**AGA**	34,334	1.18
	**AAG**	109,717	1.37		**AGG**	31,972	1.10
Asp	**GAU**	85,457	1.00	Gly	GGU	32,910	0.77
	GAC	85,476	1.00		**GGC**	49,200	1.15
Glu	GAA	72,247	0.78		**GGA**	47,874	1.12
	**GAG**	112,061	1.22		GGG	40,586	0.95

N: The number of codons; the frequently used codons of *G. duodenalis* are displayed in bold.

**Table 2:** 15 most frequently used codons of *G. duodenalis* highly expressed VSPs genes.

Codon Amino Acid Fraction Frequency Number
**UGC** Cys 0.730 88.230 1042
**AAG** Lys 0.760 60.203 711
**GGC** Gly 0.367 40.898 483
**GAC** Asp 0.575 35.309 417
**AAC** Asn 0.679 34.208 404
**GCC** Ala 0.299 32.769 387
**ACG** Thr 0.325 32.769 387
*UGU* Cys 0.270 32.684 386
**GAG** Glu 0.661 30.821 364
*GGA* Gly 0.268 29.890 353
**ACC** Thr 0.284 28.620 338
**GCG** Ala 0.257 28.196 333
**GGG** Gly 0.242 26.926 318
GAU Asp 0.425 26.080 308
**AGC** Ser 0.367 26.080 308

**Bold**, frequently used codons ended with G or C; Italics, frequently used codons ended with A or U.

**Table 3:** The codon usage table of Giardia intestinalis was obtained from the Codon Usage Database via Optimizer software.

C	AA	FRA.	FRE.	N	C	AA	FRA.	FRE.	N	C	AA	FRA.	FRE.	N	C	AA	FRA.	FRE.	N
UUU	F	0.44	14.6	2723	UCU	S	0.23	17.7	3293	UAU	Y	0.44	14.3	2658	UGU	C	0.35	12.6	2349
UUC	F	0.56	18.9	3516	UCC	S	0.19	14.1	2632	UAC	Y	0.56	18.5	3448	**UGC**	C	0.65	23.8	4430
UUA	L	0.07	5.9	1095	UCA	S	0.13	9.9	1849	UAA	*	0.53	0.7	137	UGA	W	0.07	0.6	108
UUG	L	0.11	10.0	1861	UCG	S	0.12	9.2	1721	UAG	*	0.47	0.7	122	UGG	W	0.93	7.4	1378
CUU	L	0.24	21.0	3091	CCU	P	0.25	11.2	2077	CAU	H	0.36	7.6	1421	CGU	R	0.16	7.9	1462
**CUC**	L	0.28	24.6	4578	CCC	P	0.27	11.9	2215	CAC	H	0.64	13.4	2493	CGC	R	0.28	13.7	2552
CUA	L	0.10	9.1	1702	CCA	P	0.24	10.5	1963	CAA	Q	0.33	12.0	2228	CGA	R	0.10	4.8	891
CUG	L	0.20	17.5	3266	CCG	P	0.23	10.2	1898	**CAG**	Q	0.67	24.5	4551	CGG	R	0.10	4.8	896
AUU	I	0.32	17.3	3217	ACU	T	0.23	14.9	2776	AAU	N	0.40	16.5	3074	AGU	S	0.12	8.9	1658
**AUC**	I	0.44	23.7	4418	ACC	T	0.27	18.1	3377	**AAC**	N	0.60	24.4	4548	AGC	S	0.22	16.4	3061
AUA	I	0.25	13.5	2505	ACA	T	0.27	17.9	3331	AAA	K	0.23	13.8	2574	AGA	R	0.16	7.7	1433
AUG	M	1.00	21.3	3961	ACG	T	0.23	15.3	2846	**AAG**	K	0.77	45.3	8433	AGG	R	0.19	9.4	1749
GUU	V	0.26	16.2	3009	GCU	A	0.23	19.3	3583	**GAU**	D	0.42	24.6	4575	GGU	G	0.17	11.6	2155
GUC	V	0.36	22.7	4229	**GCC**	A	0.30	25.3	4707	**GAC**	D	0.58	33.6	6253	GGC	G	0.34	23.1	4291
GUA	V	0.13	8.0	1483	GCA	A	0.28	23.2	4318	GAA	E	0.31	18.9	3516	GGA	G	0.25	16.8	3128
GUG	V	0.25	15.6	2895	GCG	A	0.19	15.9	2965	**GAG**	E	0.69	41.5	7719	GGG	G	0.23	15.7	2917

**Bold**, 10 most frequently used codons. **C**, Codon; **AA**, amino acid; **FRA.**, fraction; **FRE**., frequency; **N**, number.

**Table 4:** Translational optimal codons of *G. duodenalis*.

AA	Codon	High RSCU (N)	Low RSCU (N)	AA	Codon	High RSCU (N)	Low RSCU (N)
Phe	UUU	0.36 (632)	1.24 (1846)	Ser	UCU	0.88 (972)	1.58 (3140)
	UUC *	1.64 (2845)	0.76 (1132)		UCC *	1.66 (1833)	0.75 (1484)
Leu	UUA	0.07 (94)	0.99 (1832)		UCA	0.27 (301)	1.20 (2392)
	UUG	0.27 (377)	0.96 (1777)		UCG *	1.13 (1249)	0.53 (1044)
	CUU	0.96 (1350)	1.31 (2421)		AGU	0.32 (353)	0.98 (1943)
	CUC *	2.96 (4153)	0.76 (1399)		AGC *	1.74 (1929)	0.96 (1915)
	CUA	0.14 (190)	1.06 (1962)	Pro	CCU	0.64 (807)	1.21 (1684)
	CUG *	1.61 (2262)	0.92 (1690)		CCC *	1.57 (1974)	0.76 (1063)
Ile	AUU	0.51 (801)	1.16 (2187)		CCA	0.38 (480)	1.43 (1992)
	AUC *	2.16 (3418)	0.73 (1369)		CCG *	1.41 (1778)	0.59 (824)
	AUA	0.33 (529)	1.11 (2088)	Thr	ACU	0.50 (729)	1.21 (2058)
Met	AUG	1.00 (2296)	1.00 (2331)		ACC *	1.30 (1914)	0.77 (1306)
Val	GUU	0.60 (1048)	1.13 (1611)		ACA	0.62 (917)	1.42 (2413)
	GUC *	2.27 (3977)	0.74 (1066)		ACG *	1.58 (2319)	0.60 (1013)
	GUA	0.17 (297)	1.11 (1590)	Ala	GCU	0.52 (1271)	1.22 (2344)
	GUG	0.96 (1687)	1.02 (1461)		GCC *	1.70 (4180)	0.77 (1487)
Tyr	UAU	0.40 (638)	1.21 (1682)		GCA	0.63 (1551)	1.47 (2826)
	UAC *	1.60 (2521)	0.79 (1095)		GCG *	1.15 (2823)	0.54 (1042)
His	CAU	0.32 (338)	1.13 (1582)	Cys	UGU	0.32 (702)	1.05 (1125)
	CAC *	1.68 (1779)	0.87 (1221)		UGC *	1.68 (3650)	0.95 (1015)
Gln	CAA	0.21 (335)	1.07 (2489)	Trp	UGG	1.00 (823)	1.00 (749)
	CAG *	1.79 (2878)	0.93 (2171)	Arg	CGU	0.57 (547)	0.87 (1036)
Asn	AAU	0.39 (763)	1.15 (2189)		CGC *	2.58 (2471)	0.75 (890)
	AAC *	1.61 (3105)	0.85 (1618)		CGA	0.25 (242)	0.93 (1105)
Lys	AAA	0.18 (548)	0.92 (2284)		CGG	0.91 (868)	0.89 (1056)
	AAG *	1.82 (5508)	1.08 (2680)		AGA	0.37 (355)	1.57 (1859)
Asp	GAU	0.45 (1310)	1.23 (3040)		AGG *	1.32 (1264)	0.99 (1168)
	GAC *	1.55 (4509)	0.77 (1913)	Gly	GGU	0.41 (833)	0.96 (1045)
Glu	GAA	0.23 (721)	1.00 (2804)		GGC *	1.87 (3831)	0.91 (987)
	GAG *	1.77 (5474)	1.00 (2795)		GGA	0.54 (1106)	1.30 (1407)
					GGG *	1.18 (2417)	0.83 (898)

Comparison of codon usage frequency between high and low expression genes of *G. duodenalis*. The optimal codons were determined by a Chi-square contingency test. * indicates that the frequency of the codons is much higher (*p* < 0.01). AA, amino acid; N, number of codons.

## Data Availability

Excluded this statement.

## Abbreviations

GC1	GC-content at the first codon positions
GC2	GC-content at the second codon positions
GC12	The average of GC1 and GC2
GC3	GC-content at the third codon positions
GC3s	Frequency of either a G or C at the third codon position of synonymous codons
